# Compression Properties and Fabrication of Closed-Cell Metal Matrix Syntactic Foams Al_2_O_3hs_/AZ91D

**DOI:** 10.3390/ma15196873

**Published:** 2022-10-03

**Authors:** Changyun Li, Erkuo Yang, Ling Tang, Yang Li, Lei Xu

**Affiliations:** 1 Faculty of Engineering, China University of Petroleum-Beijing at Karamay, Karamay 834000, China; 2 School of Materials Science and Engineering, Henan Polytechnic University, Jiaozuo 454003, China

**Keywords:** hollow spheres, Al_2_O_3_, metal matrix syntactic foams, powder metallurgy

## Abstract

Closed-cell metal syntactic foam is a new material consisting of hollow spheres embedded in metal matrix syntactic foams. These foams have good physical and mechanical properties and are increasingly used worldwide in industrial and high-tech fields. Magnesium matrix syntactic foams containing hollow Al_2_O_3_ spheres ((Al_2_O_3hs_)/AZ91D) were successfully fabricated by hot press sintering at different temperatures. The fabrication of Al_2_O_3hs_/AZ91D and the effect of sintering temperature on the microstructure and properties are reported in this paper. Additionally, sandwiched magnesium matrix syntactic foams were prepared by placing magnesium plates on both sides of the syntactic foam. Some Al_2_O_3hs_ particles became filled with matrix particles during preparation. Thus, the actual density was greater than the theoretically calculated value and increases with increasing sintering temperature. Above 723 K, a brittle phase MgAl_2_O_4_ formed in Al_2_O_3hs_/AZ91D. The quasistatic and dynamic compressive strengths of Al_2_O_3hs_/AZ91D first increased and then decreased with increasing sintering temperature, and the maximums were 162 MPa and 167.87 MPa, respectively. Thus, this paper reports a new strategy for the controlled preparation of metal matrix syntactic foams with predetermined porosity. The results show that this strategy improved the performance of lightweight and high-strength syntactic foam materials and shows potential for further research.

## 1. Introduction

Metal matrix syntactic foams (MMSFs) are synthesized by dispersing hollow particles in a metallic matrix. The hollow particles provide these materials with closed-cell structures and make them more lightweight compared to the matrix alloy [1,2]. The properties of the two materials are combined in MMSFs, including high specific strength and specific stiffness, low density, outstanding compressive properties, and excellent energy absorption capability compared to conventional open- and/or closed-cell metallic foams [3,4,5]. As a consequence, MMSFs are used as energy absorbers, e.g., bumpers to protect automobiles against impacts and crashes, protective skins on military vehicles, and hull materials for deep sea applications and aeronautics [4].

MMSFs can be produced by stir casting [6,7,8], pressure infiltration [5,9,10,11], powder metallurgy (PM) [4,12,13,14], and so on. Pressure infiltration and stir casting are common ways to fabricate MMSFs in which the matrix is in the liquid stages [2]. During stir casting, the matrix material is melted and preheated, and cenospheres are added in relatively small quantities under continuous stirring [11,15]. Although this method is relatively simple, there is the potential for the cenospheres to fracture during mixing. Thus, this method is only suitable for producing MMSFs with a low volume fraction of cenospheres. Compared to the theoretical volume fraction, a lower volume fraction of filling material can always be achieved. The disadvantages of pressure infiltration are the need for complex production equipment, difficult process of producing MMSFs with different volume fractions of cenospheres, and potential for cenospheres to be crushed or become filled with the matrix as the infiltration pressure exceed their fracture strength [11,15].

The powder metallurgy method is the most suitable for fabricating MMSFs [4,12,13,16,17]. Its most important advantage is its low processing temperature, a condition not shared by the melting technique which prevents the reaction of the matrix with the reinforcement to form undesired phases. Moreover, hollow particles are easily and uniformly distributed throughout the matrix. Guo and Rohatgi [18] first attempted to produce and evaluate MMSFs prepared by PM while using fly ash particles as a reinforcement. Their study demonstrated that the proper selection of the compacting pressure played a vital role in the PM of cenosphere MMSFs. Neville and Rabiei [1] used steel particles and cenospheres as reinforcements to produce a new type of MMSF with better mechanical properties than many existing foams.

Common filling materials [19] include fly ash [10,20,21,22,23], SiC hollow spheres [24], cenospheres [4,25,26,27,28], glass microspheres [29,30,31,32], expanded perlite [33,34,35], and pumice [36]. However, there have been few studies on the use of Al_2_O_3hs_ as filling material to fabricate MMSFs.

In this study, Al_2_O_3hs_/AZ91D syntactic foams were sintered at 663, 693, 723, and 753 K by the hot press sintering method, and the structural properties of Al_2_O_3hs_/AZ91D were investigated by using the optical microscopy (OM), scanning electron microscopy (SEM), X-ray diffraction (XRD), and energy dispersive X-ray spectroscopy (EDS) techniques. To further improve the strength of the syntactic foams, the interface between the syntactic foams and magnesium plates added on both sides of the MMSFs were studied. Here, the controlled preparation of metal matrix syntactic foams with predetermined porosity was reported to strengthen the lightweight and high-strength syntactic foam materials.

## 2. Materials and Methods

### 2.1. Fabrication of the Al_2_O_3hs_/AZ91D Syntactic Foams

**Figure 1:**
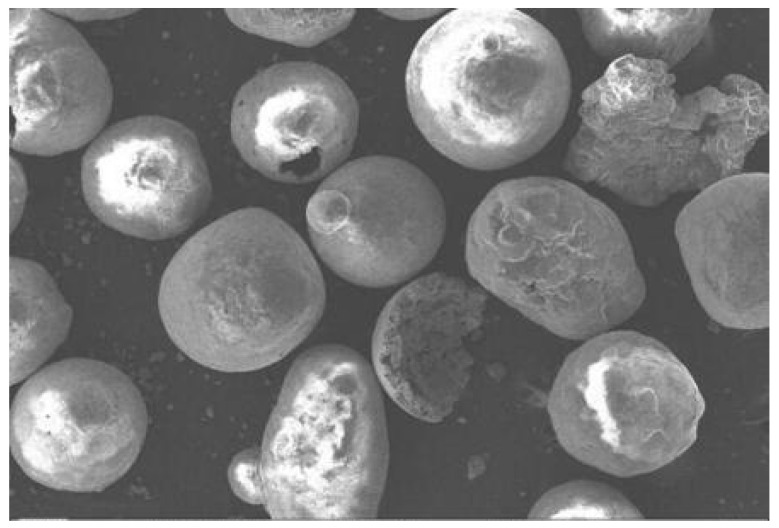
Micrograph of the Al_2_O_3hs_.

**Figure 2:**
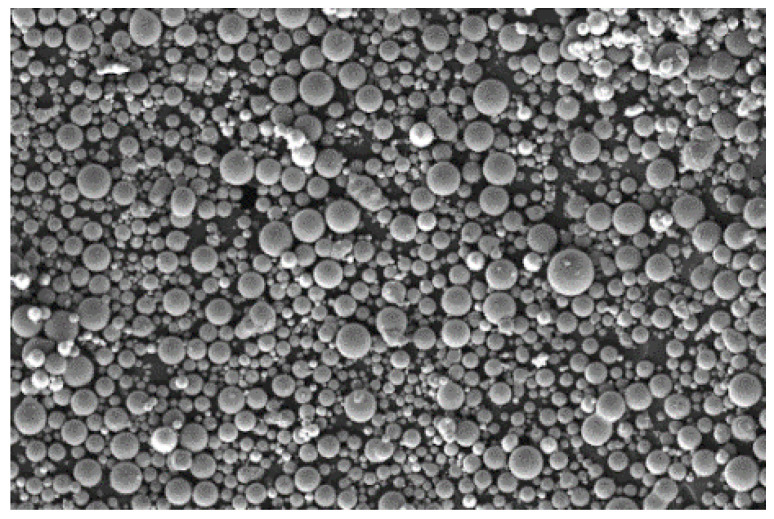
SEM micrograph of the AZ91D matrix.

In this study, Al_2_O_3_ hollow spheres (Al_2_O_3hs_, α-Al_2_O_3_ crystal, 600–850 μm, wall thickness of about 40 μm, Ruizi Technology Co., Ltd. in Beijing, China) and commercial AZ91D (1.69 g/cm^3^, ≤325 mesh, Weihao New Materials Co., Ltd. in Tangshan, China) were used as the filling and matrix materials, respectively, to prepare lightweight syntactic foam. The microstructures of Al_2_O_3hs_ and AZ91D are shown in Figure 1 and Figure 2. A few spherical shells with small holes and fragments were observed. The main components of AZ91D are listed in Table 1. D_V_ (10), D_V_ (50), and D_V_ (90) are listed in Table 2, and D_V_ (50) = 758 μm.

Syntactic powders with 60 vol% Al_2_O_3hs_ were mixed in a high-speed vibrating ball mill eccentric swing for 30 min without ball, to avoid crushing the Al_2_O_3hs_. Then, hybrid powders were loaded into a cylindrical graphite mold with an interior hole (diameter 30 mm) and hot pressed at a set temperature (663, 693, 723, 753 K) and pressure of 25 MPa for 1 h at a heating rate of 10 K/min in Ar-protective atmosphere. The hot press sample was cooled naturally in the furnace (Chenhua Science Technology Corp., Ltd. in Shanghai, China).

### 2.2. Characterization Methods

**Figure 3:**
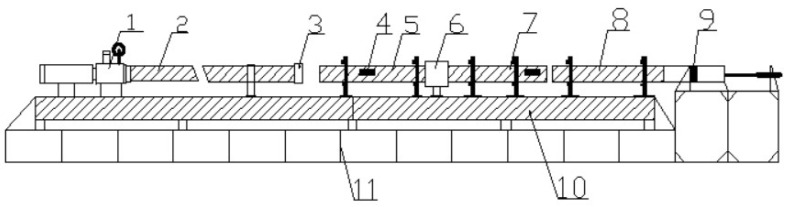
Schematic diagram of the SHPB setup: 1—launcher, 2—launch gun barrel, 3—speedometer, 4—strain gauge, 5—incident bar, 6—sample platform, 7—transmitted bar, 8—damping bar, 9—damper, 10—slideway, and 11—base.

Specimens for the microstructural observations were prepared using standard metallographic procedures, including grinding, polishing, and etching. An Olympus optical microscope was used to observe the Al_2_O_3hs_/AZ91D syntactic foam microstructures and the distribution of Al_2_O_3hs_. The microstructural properties were characterized using an X-ray diffractometer (Rigaku SmartLab, in Osaka, Japan) with a Cu Ka radiation source (I = 1.54056 A) at a scanning speed of 5°/min in the 2θ range of 10–90° and a scanning electron microscope (FEI Quanta 250 FEG; in Hillsboro, USA) equipped with an Oxford Instrument energy dispersive X-ray detector (Bruker Quantax 200 XFlash 6|30; in Hillsboro, OR, USA). The specimens were freeze-fractured and sputter-coated with gold before SEM was performed. The quasistatic compressive strength was measured by a universal testing machine (CMT5205, MTS Systems (China) Co. Ltd., Shenzhen, China) with a constant nominal crosshead speed of 1 mm/min, according to the international standard ISO13314. Dynamic compression was undertaken using a split Hopkinson pressure bar (SHPB) prepared by Luoyang Levi Technology, as shown in Figure 3, and the air pressure was 0.5–0.75 MPa. The lengths of the striker, incident bar, and transmission bar were 1000 mm, 2500 mm, and 2500 mm, respectively. The specimen was sandwiched between the incident bar and transmission bar. Moreover, since the stress wave transmitted from the sample into the transmission bar was too small, the semiconductor strain gages were adopted to record the weak transmission waves, with the sensitivity coefficient of 2.08–2.11. The strain histories recorded by strain gauges fixed on the incident and transmission bars were converted to the stress–strain curve. The energy absorption efficiency was determined to investigate the variation in compressive deformation resistance using Equation (1):

(1) $$We=\frac{\int_{0}^{0.5}\delta d\varepsilon }{0.5\times \sigma_{max}}$$

where *σ_max_* is the maximum stress observed up to a strain of *ε* = 0.5.

## 3. Results

### 3.1. Optical Macrostructure and Microstructure of the Al_2_O_3hs_/AZ91D Syntactic Foam

**Figure 4:**
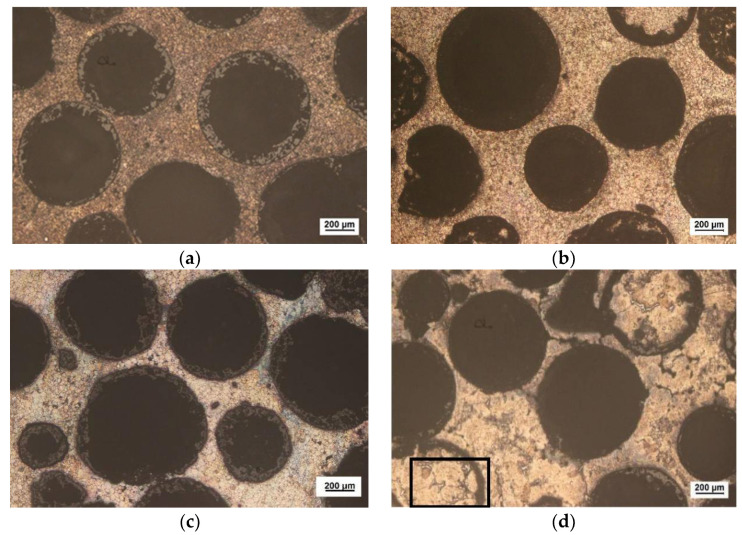
Microstructure of Al_2_O_3hs_/AZ91D prepared at different temperatures: (**a**) 663 K, (**b**) 693 K, (**c**) 723 K, and (**d**) 753 K.

**Figure 5:**
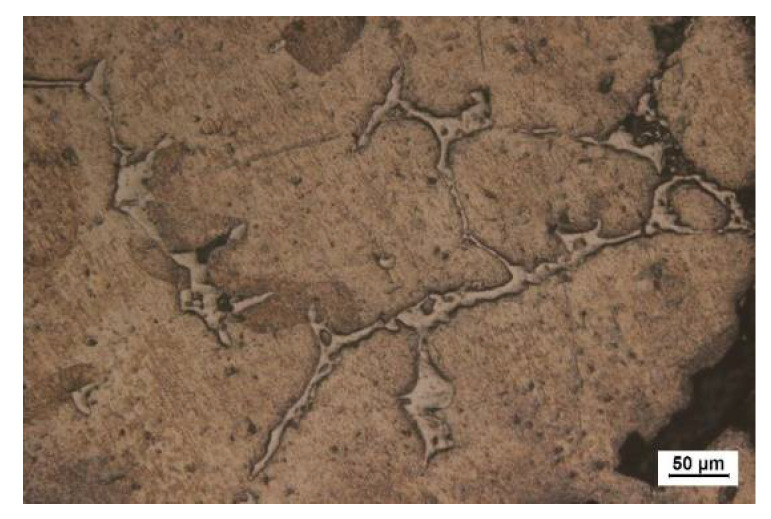
Enlarged image of the area in the box in Figure 4d.

**Figure 6:**
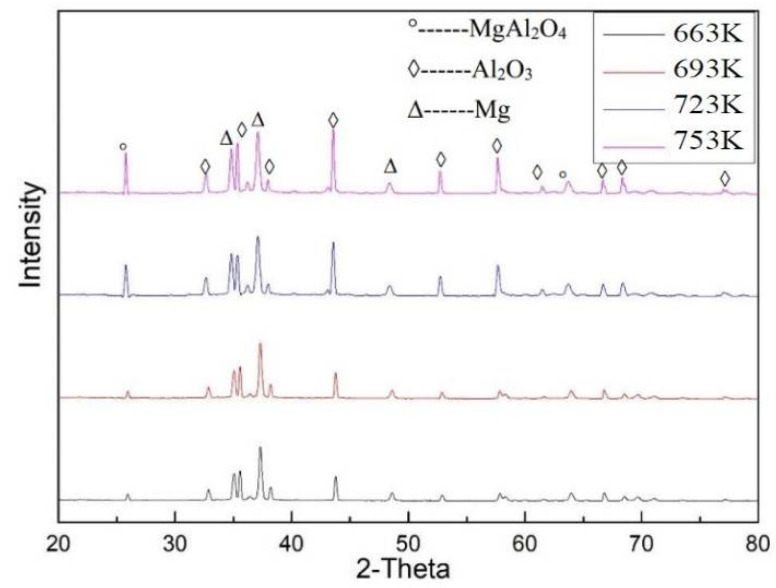
XRD patterns of Al_2_O_3hs_/AZ91D.

**Figure 7:**
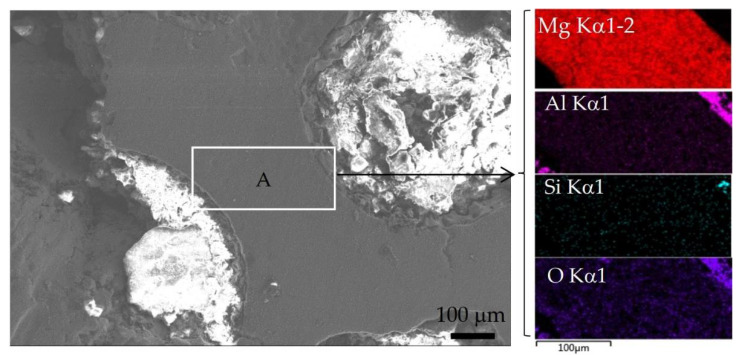
Local microstructure of Al_2_O_3hs_/AZ91D prepared at 693 K and its elemental map.

Figure 4 shows optical metallographic pictures of 60 vol% Al_2_O_3hs_/AZ91D prepared at a sintering pressure of 25 MPa and sintering temperatures of 663, 693, 723, and 753 K. As shown in Figure 4, the hollow spheres were round and evenly distributed in the matrix. In this group of experiments, Al_2_O_3hs_ with particle sizes of mostly 425–600 μm was observed. The particle size fluctuation shown in Figure 4a,b was small. In Figure 4c, some small hollow spheres of Al_2_O_3hs_ were visible, and the particle size fluctuated greatly due to the normal size distribution of the Al_2_O_3hs_ hollow spheres. A very small number of small Al_2_O_3hs_ hollow spheres was observed, as shown in Figure 4. Figure 4d shows that the fluctuation in the size was small, and some Al_2_O_3hs_ fragments were observed. Figure 4c, d are compared with Figure 4a,b at sintering temperatures of 723 and 753 K, and the results show that the wall thickness of Al_2_O_3hs_ was uneven and fluctuated greatly due to the interfacial reaction between Al_2_O_3hs_ and the matrix. Figure 4a,b show that at 663 and 693 K, the size fluctuation of the matrix and the sizes of the Al_2_O_3hs_/AZ91D are small. Compared with Figure 4a,b, the matrix particles in Al_2_O_3hs_/AZ91D increases in size when prepared at 663 and 693 K. Additionally, in Figure 4d, some of the hollow spheres are filled with matrix particles. This is due to the incomplete shell or pores in the hollow spheres. During the powder mixing process, matrix particles filled the hollow spheres and were then sintered by hot pressing. In addition, bright dendritic precipitates were observed in the cavities of hollow spheres filled with matrix particles, as shown in Figure 5. Moreover, there are some bright precipitates in the matrix outside the hollow spheres. Figure 6 shows the XRD results. According to the previous literature [37], SiO_2_ and Al_2_O_3_, the major constituents of the hollow spheres, can react with Mg and Al, according to Equations (2) and (3), forming the MgAl_2_O_4_ phase. Furthermore, the distributions of Al, Mg, Si, and O at 693 K are shown in Figure 7. Figure 7 shows that elemental diffusion occurred between the Al_2_O_3hs_ and AZ91D matrix, and there is a narrow transition layer on the contact surface. Besides that, the main elements are distributed uniformly in the matrix, indicating no other second phase forming.

(2) $$3\mathrm{Mg}\left(l\right)+{\mathrm{Al}}_{2}O_{3}\left(s\right)\to 3{\mathrm{MgAl}}_{2}O_{4}\left(s\right)+2\mathrm{Al}\left(l\right)\;∆G_{1000K}=-2576\;{\mathrm{kJ}\;\mathrm{mol}}^{-1}$$

(3) $$\mathrm{Mg}\left(l\right)+2\mathrm{Al}\left(l\right)+2{\mathrm{SiO}}_{2}\left(s\right)\to {\mathrm{MgAl}}_{2}O_{4\;}\left(s\right)+2\mathrm{Si}\left(s\right)\;∆G_{1000K}=-440.7\;{\mathrm{kJ}\;\mathrm{mol}}^{-1}$$

(l = liquid and s = solid).

### 3.2. Density of the Al_2_O_3hs_/AZ91D syntactic foam

**Figure 8:**
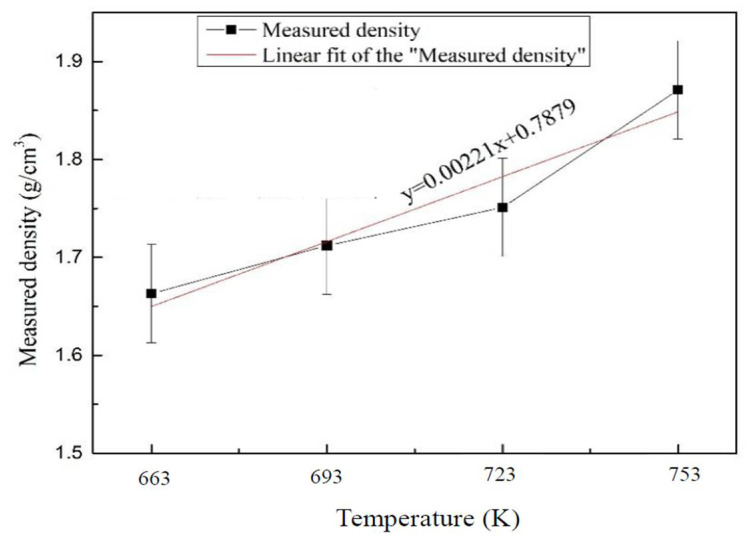
Density of the Al_2_O_3hs_/AZ91D syntactic foams.

Figure 8 shows the density of Al_2_O_3hs_/AZ91D syntactic foam at temperatures of 663, 693, 723, and 753 K. The actual density of Al_2_O_3hs_/AZ91D syntactic foam was positively correlated with the sintering temperature. The actual density of the Al_2_O_3hs_/AZ91D sintered at 663 K was 1.661 g/cm^3^. Thus, the higher the temperature was, the stronger the plastic mobility of the AZ91D matrix. Under pressure, the matrix flow filled the pores in the syntactic foams, thus increasing their density. With increasing temperature, the density increased gradually. The actual density of the Al_2_O_3hs_/AZ91D sintered at 753 K was 1.861 g/cm^3^. The linear equation obtained by fitting the data for the actual density vs. temperature was:

*y* = 0.00221*x* + 0.7879 (4)

where *y* is the actual density of the MMSFs and *x* is the sintering temperature of the MMSFs.

### 3.3. Compressive Properties of the Al_2_O_3hs_/AZ91D Syntactic Foam

#### 3.3.1. The Quasistatic Compressive Properties of the Al_2_O_3hs_/AZ91D Syntactic Foam

**Figure 9:**
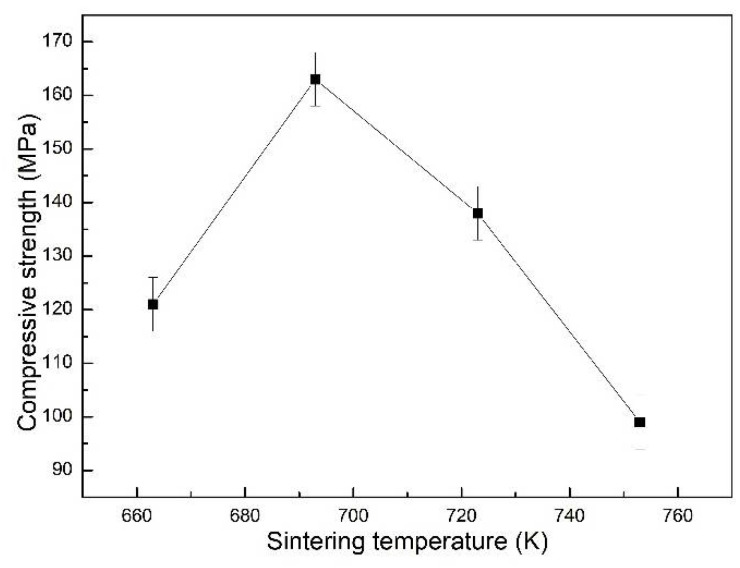
Quasistatic compressive strength of the Al_2_O_3hs_/AZ91D syntactic foams.

Figure 9 shows the quasistatic compressive strength of the Al_2_O_3hs_/AZ91D syntactic foam at different sintering temperatures. With increasing sintering temperature, the compressive strength of Al_2_O_3hs_/AZ91D first increased and then decreased, and the maximum value was obtained at 693 K. The quasistatic compressive strength of the Al_2_O_3hs_/AZ91D syntactic foam sintered at 663 K reached 120 MPa due to the weak bonding at relative low temperatures. With increasing sintering temperature, the compressive strength of the Al_2_O_3hs_/AZ91D syntactic foam gradually increased. The maximum value was 167 MPa for Al_2_O_3hs_/AZ91D sintered at 693 K. With a further increase in the sintering temperature, the compressive strength of the Al_2_O_3hs_/AZ91D syntactic foam decreased gradually to 97 MPa for Al_2_O_3hs_/AZ91D sintered at 753 K. According to the XRD pattern in Figure 6, many brittle MgAl_2_O_4_ phases formed at 723 and 753 K. Under loading, the brittle phase was the first to break, which led to a decrease in the quasistatic compressive strength of the Al_2_O_3hs_/AZ91D syntactic foam prepared at 723 and 753 K. Compared with the data in the literature [4,6,25,37,38,39,40,41,42,43,44,45,46,47], the performance of the present Al_2_O_3hs_/AZ91D syntactic foam is much better.

**Figure 10:**
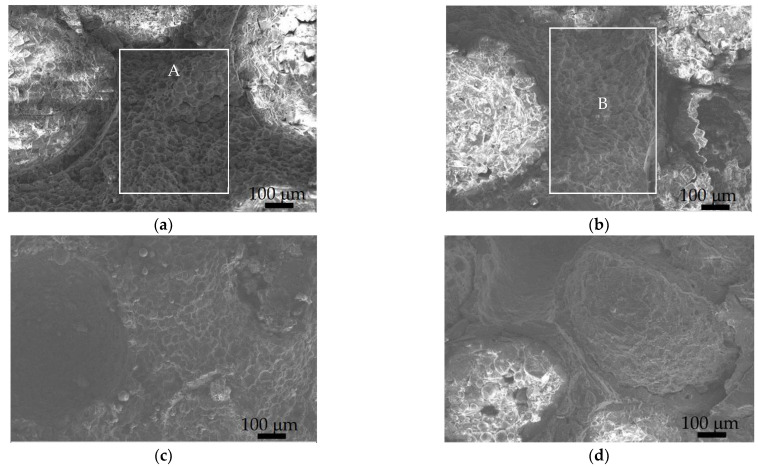
Quasistatic compressive fracture morphology of Al_2_O_3hs_/AZ91D prepared at different sintering temperatures: (**a**) 663 K, (**b**) 693 K, (**c**) 723 K, and (**d**) 753 K.

Figure 10 shows the quasistatic compression fracture morphology of the Al_2_O_3hs_/AZ91D syntactic foams prepared at different sintering temperatures of 663 K, 693 K, 723 K, and 753 K. Figure 10a,b show that for the Al_2_O_3hs_/AZ91D sintered at 663 K and 693 K, many Mg particles in the fracture morphology were polygonal in the rectangle remarked as A, B area, and each surface of the polygon was relatively flat with a large number of dimples. The Al_2_O_3hs_ microspheres in the matrix broke from the lower section, leaving residual Al_2_O_3hs_ microspheres. With an increase in sintering temperature, the Al_2_O_3hs_/AZ91D sintered at 723 K and 753 K retained the small dimples caused by the many Mg particles in the fracture morphology. However, the color of the dimple surface was brighter due to the brittle phase MgAl_2_O_4_ in the Al_2_O_3hs_/AZ91D syntactic foam formed during the hot press sintering process.

#### 3.3.2. Dynamic Compact Properties of the Al_2_O_3hs_/AZ91D Foams

The specific values of the compression properties are shown in Table 3, according to Equation (1). The yield strength and compressive strength of the Al_2_O_3hs_/AZ91D prepared at 663 K were 143.5 MPa and 139.5 MPa, respectively. The yield strength and compressive strength reached maximum values of 167.87 MPa and 156.10 MPa, respectively, at 693 K. At a sintering temperature of 753 K, the yield strength and compressive strength of Al_2_O_3hs_/AZ91D exhibited their minimum values of 112.5 MPa and 98.85 MPa, respectively.

**Figure 11:**
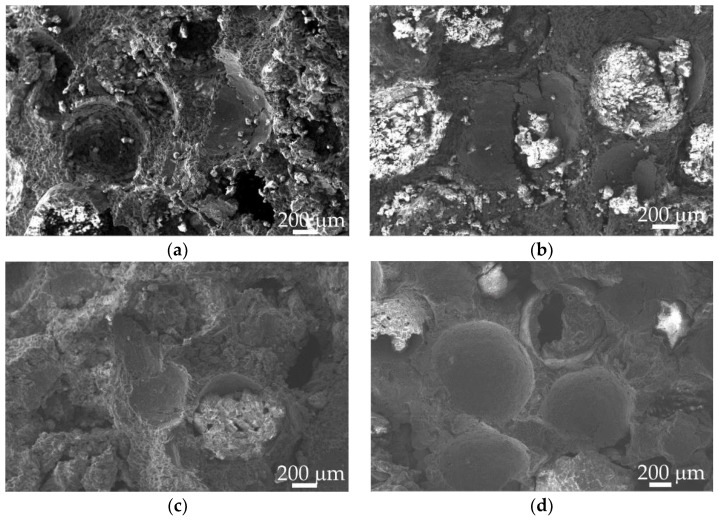
Fracture morphology at cross section of the Al_2_O_3hs_/AZ91D syntactic foams prepared at different sintering temperatures after dynamic compact: (**a**) 663 K, (**b**) 693 K, (**c**) 723 K, and (**d**) 753 K.

Figure 11 shows the fracture morphology of the Al_2_O_3hs_/AZ91D syntactic foams sintered at different temperatures. As shown in Figure 11a, for the Al_2_O_3hs_/AZ91D sintered at 663 K, some Al_2_O_3hs_ microspheres were removed and holes appeared in the matrix; some Al_2_O_3hs_ microspheres were broken in the middle, and fragments of the damaged Al_2_O_3hs_ microspheres were left. Additionally, as the matrix broke, many dimples remained. As shown in Figure 11b, at 693 K, as the Al_2_O_3hs_/AZ91D bore the load, a crack expanded to the connection between the matrix and Al_2_O_3hs_ microspheres. Due to the weak bonding between the matrix and Al_2_O_3hs_ microspheres, the crack bypassed the Al_2_O_3hs_ microspheres and pulled them out of the matrix. In the Al_2_O_3hs_/AZ91D sintered at 723 K and 753 K, many dimples remained in the matrix, and the surface color of the dimples was bright, which was mainly related to the brittle MgAl_2_O_4_ phase formed during the hot press sintering process. Therefore, during the high-frequency impact of Al_2_O_3hs_/AZ91D, the fracture mechanism of the matrix of Al_2_O_3hs_/AZ91D syntactic foam was mainly ductile fracture, while that of the microspheres was brittle fracture.

### 3.4. Research on the Preparation and Properties of Sandwiched Magnesium Matrix Syntactic Foams (Plates Added)

**Figure 12:**
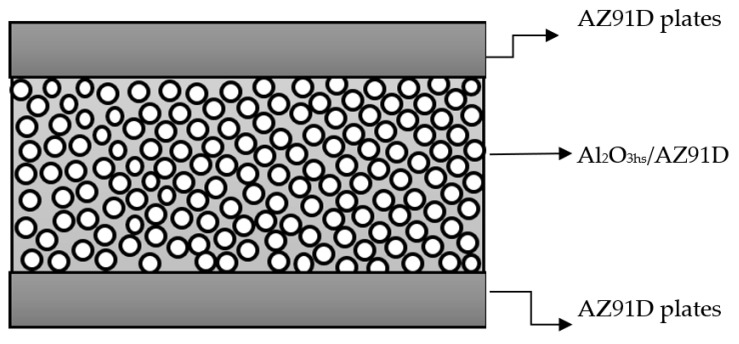
Schematic diagram of the sandwiched magnesium matrix syntactic foams.

Based on Section 3.1, Section 3.2 and Section 3.3, AZ91D magnesium plates (Guangzhou Hongqi Metal Co., Ltd., Guangzhou, China) with different thicknesses were placed on both sides of the Al2O3hs/AZ91D syntactic foam layers to prepare sandwiched Mg matrix syntactic foams at 693 K and 20 MPa. The preparation process is shown in Table 4, and a schematic diagram is shown in Figure 12, followed by the influence of the thickness of both sides on the density and compressive strength of the sandwiched syntactic foams.

**Figure 13:**
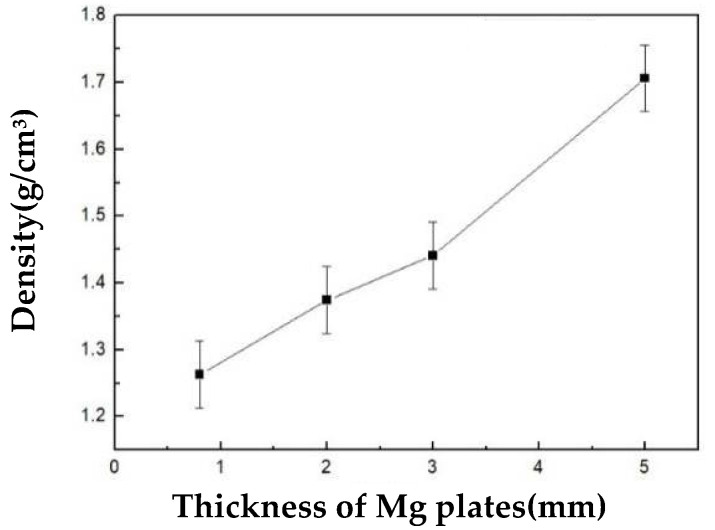
Density of the sandwiched magnesium matrix syntactic foams with different end plate thicknesses.

Figure 13 shows the relationship between the density of the sandwiched magnesium matrix syntactic foam and the thickness of both end plates. As shown in Figure 13, the density of the sandwiched syntactic foam material was 1.263 g/cm^3^ with a thickness of 0.8 mm. The strength of the sandwiched syntactic foam increased with increasing thickness of both end plates. As the thickness of both plates increased to 5 mm, the density of the sandwiched syntactic foam reached 1.706 g/cm^3^. The density depends on the properties of the sandwiched syntactic foam. The density of the AZ91D magnesium plate on both sides is higher than that of the sandwiched structure, but the inner part of the sandwich retained the same structure, density, and other properties. With the increase in thickness of both plates, the overall density of the sandwiched syntactic foam gradually increased.

**Figure 14:**
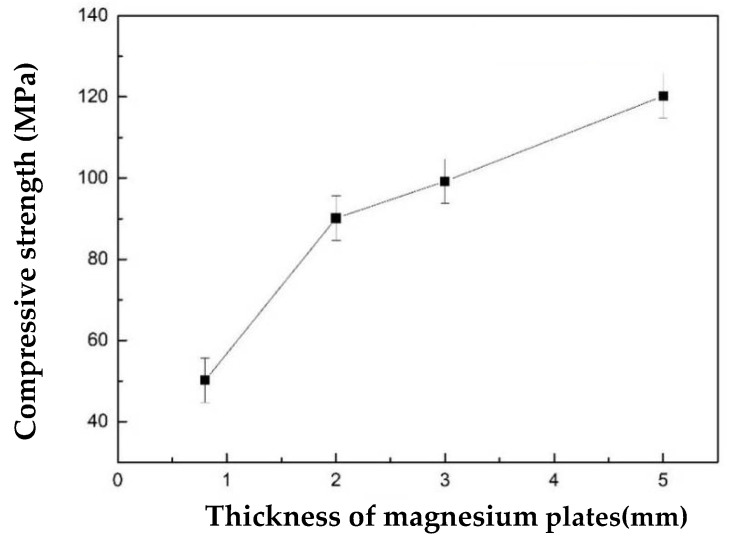
Quasistatic compression of sandwiched magnesium syntactic foam with different thicknesses of the two side plates.

Figure 14 is a diagram of the relationship between the quasistatic compression of the sandwiched magnesium matrix syntactic foam and the thickness of both plates. According to Figure 14, the quasistatic compressive strength of the sandwiched syntactic foam was 45 MPa when the thickness of the two sides was 0.8 mm. The strength of the sandwiched syntactic foam increased with increasing thickness of both plates. When the thickness of both plates was increased to 5 mm, the strength of the sandwiched syntactic foam reached 120.2 MPa, which was approximately three times that when the plates were 0.8 mm thick, and twice that of VF460 (62.7 MPa) and RR30 (59.4 MPa) [48]. However, the strength of the sandwiched syntactic foam in this study differed from the ideal strength. Thus, this paper reports a new strategy for the controlled preparation of metal matrix syntactic foams with predetermined porosity and improvement of the performance of syntactic foam materials for further research.

Gupta [48] and Orbulov [49] studied three typical fracture modes (A’, B’, and C’) of MMSFs. The first two (A’ and B’) were related to the formation of compression cones at the top and bottom of the specimen at approximately 30° and 45°, respectively. A shear plane is formed at the top of the wall. The third mechanism (C’) was related to the initiation of failure along some weak planes transverse to the applied compressive load.

**Figure 15:**
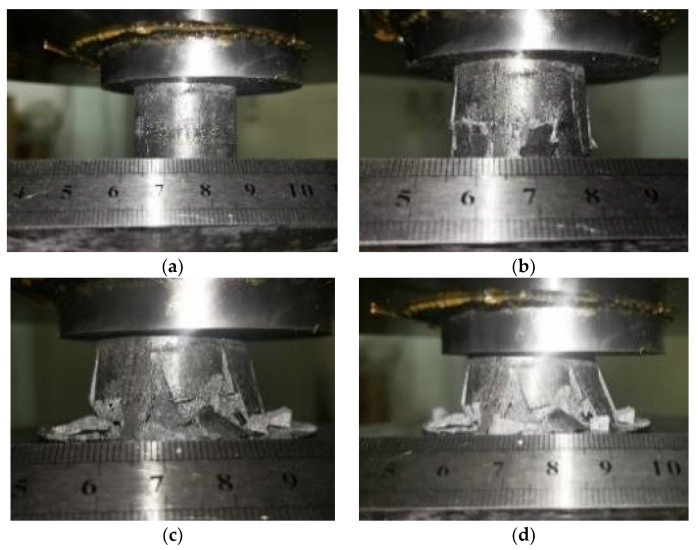
Quasistatic compression process diagram. (**a**) Initial stage; (**b**,**c**) Intermediate stage; (**d**) Final stage.

Figure 15 shows the characteristics of longitudinal cracks at the bottom of the specimen during compression. With an increase in deformation, the cracks in the syntactic foams started from the bottom, and the outer wall separated from the main body, exposing the inner core of the material (Figure 15b,c). This behavior was related to the uneven density distribution of the sandwiched syntactic foams. The density of the AZ91D magnesium plates at the bottom of the sandwiched syntactic foam was greater than that of the Al_2_O_3hs_/AZ91D syntactic foam. The density of the sandwiched Al_2_O_3hs_/AZ91D syntactic foam was less than that of the Al_2_O_3hs_/AZ91D syntactic foam. Some studies have shown that contact between hollow microspheres is beneficial to the growth of syntactic foams at 30° and 45°, where fracture cracks form [5,50]. In this case, when under strain, a 30° shear plane formed inside the Al_2_O_3hs_/AZ91D syntactic foam, indicating brittle fracture of the material. As compression continued, the fracture of the material gradually progressed from the bottom to the top, and the dense part of the sample (with uniform porosity) deformed along with the appearance of a 30° shear plane. In this process, Al_2_O_3hs_ bore the load and gradually broke, layer by layer, while absorbing a large amount of energy. As a result, the upper region of the specimen was nearly unaffected. Due to the low porosity of these regions and sufficient bonding between the matrix and Al_2_O_3hs_ particles, the best deformation response was obtained.

## 4. Conclusions

In this study, Al_2_O_3hs_/AZ91D was successfully prepared by a sintering method involving hot pressing. The effects of sintering temperature on the microstructure and mechanical properties of Al_2_O_3hs_/AZ91D syntactic foam were studied. The main conclusions are as follows: Al_2_O_3hs_ was distributed uniformly in the AZ91D matrix, and some Al_2_O_3hs_ microspheres were filled with matrix particles. The actual density increases with the sintering temperature. With increasing sintering temperature, a brittle MgAl_2_O_4_ phase is formed in Al_2_O_3hs_/AZ91D. In quasistatic compression and high-speed dynamic impact tests, the compressive strength of Al_2_O_3hs_/AZ91D first increased and then decreased with increasing sintering temperature. The matrix of the Al_2_O_3hs_/AZ91D syntactic foam mainly underwent ductile fracture, while Al_2_O_3hs_ underwent brittle fracture. Cracks extended around the Al_2_O_3hs_ microspheres and left a large number of exposed Al_2_O_3hs_ microspheres or vacancies in the fracture morphology. The sintering temperature of 693 K was the optimum preparation condition for Al_2_O_3hs_/AZ91D. Regarding the sandwiched magnesium matrix syntactic foam, as the thickness of both side plates was gradually increased, the overall density and strength increased. In the quasistatic compression process, the fracture of the sandwiched Mg-based syntactic foam started from the weak interface at the bottom near the magnesium plate and moved gradually towards the top. The outer wall of the sandwich layer was separated from the foam body (core) and then cracking proceeded from the bottom to the top.

Compared with the substantial progress in technologies for traditional open-cell syntactic foam composites by development of foaming methods and foaming agents, research on closed-cell syntactic foams has progressed slowly. At present, the fabrication of lightweight and high-strength syntactic foams using state-of-the-art technology is the most critical task for broad commercial applications. Future studies should focus on fundamental systematic engineering research, such as the controlled preparation of syntactic foams with predetermined porosity, according to the need for practical strength. Specifically, lightweight and high-strength syntactic foams are another opportunity to meet requirements for net zero carbon emissions. Therefore, using advanced design concepts for the controlled preparation of syntactic foams with predetermined porosity and greater mechanical strength will be a promising area for future work.

## Figures and Tables

**Table 1:** Composition of the AZ91D alloy (mass fraction, %).

Al	Cu	Fe	Mn	Ni
8.90	0.0006	0.0112	0.19	0.0030
**Si**	**Zn**	**Be**	**Impurities**	**Mg**
0.0030	0.0030	0.00098	0.001	Bal.

**Table 2:** Particle size distribution of Al_2_O_3hs_.

Item	Category	D_V_ (10)/μm	D_V_ (50)/μm	D_V_ (90)/μm
Al_2_O_3hs_	Volume fraction	575	758	1010

**Table 3:** Compressive properties of Al_2_O_3hs_/AZ91D.

Temperature/°C	Yield Strength/MPa	Compressive Strength/MPa	Strain/%	Energy Absorption (*ε* = 2%)/MJ·mm^−3^	Energy Absorption (*ε* = 6%)/MJ·mm^−3^
663	143.5	139.5	7	4.18	---
693	167.87	156.10	15	8.54	10.07
723	135.6	132.5	12	5.35	8.83
753	112.5	98.85	9	3.28	5.53

**Table 4:** Preparation of the sandwiched magnesium matrix syntactic foams.

No.	Volume Fraction/%	Sintering Pressure/MPa	Sintering Temperature/°C	Thickness of the Mg Plates/mm
#1	70	15	573 K /1 h–693 K /1 h	0.8
#2	70	15	573 K /1 h–693 K /1 h	2
#3	70	15	573 K /1 h–693 K /1 h	3
#4	70	15	573 K /1 h–693 K /1 h	5

## Data Availability

Not applicable.
